# *Nuttalliella namaqua*: A Living Fossil and Closest Relative to the Ancestral Tick Lineage: Implications for the Evolution of Blood-Feeding in Ticks

**DOI:** 10.1371/journal.pone.0023675

**Published:** 2011-08-17

**Authors:** Ben J. Mans, Daniel de Klerk, Ronel Pienaar, Abdalla A. Latif

**Affiliations:** 1 Parasites, Vectors and Vector-Borne Diseases, Agricultural Research Council-Onderstepoort Veterinary Institute, Onderstepoort, South Africa; 2 The Department of Veterinary Tropical Diseases, University of Pretoria, Pretoria, South Africa; Universidade Federal do Rio de Janeiro, Brazil

## Abstract

Ticks are monophyletic and composed of the hard (Ixodidae) and soft (Argasidae) tick families, as well as the Nuttalliellidae, a family with a single species, *Nuttalliella namaqua*. Significant biological differences in lifestyle strategies for hard and soft ticks suggest that various blood-feeding adaptations occurred after their divergence. The phylogenetic relationships between the tick families have not yet been resolved due to the lack of molecular data for *N. namaqua*. This tick possesses a pseudo-scutum and apical gnathostoma as observed for ixodids, has a leathery cuticle similar to argasids and has been considered the evolutionary missing link between the two families. Little knowledge exists with regard to its feeding biology or host preferences. Data on its biology and systematic relationship to the other tick families could therefore be crucial in understanding the evolution of blood-feeding behaviour in ticks. Live specimens were collected and blood meal analysis showed the presence of DNA for girdled lizards from the Cordylid family. Feeding of ticks on lizards showed that engorgement occurred rapidly, similar to argasids, but that blood meal concentration occurs via malpighian excretion of water. Phylogenetic analysis of the 18S nuclear and 16S mitochondrial genes indicate that *N. namaqua* grouped basal to the main tick families. The data supports the monophyly of all tick families and suggests the evolution of argasid-like blood-feeding behaviour in the ancestral tick lineage. Based on the data and considerations from literature we propose an origin for ticks in the Karoo basin of Gondwanaland during the late Permian. The nuttalliellid family almost became extinct during the End Permian event, leaving *N. namaqua* as the closest living relative to the ancestral tick lineage and the evolutionary missing link between the tick families.

## Introduction

Ticks (Ixodida) are composed of three main families, the hard ticks (Ixodidae∼700 species), the soft ticks (Argasidae∼200 species) and the Nuttalliellidae (monotypic) [1]–[2]. Genetic and morphological data indicates that the hard and soft tick families are monophyletic to the exclusion of all other mites [1], [3]–[5], suggesting that a blood-feeding lifestyle evolved within the ancestral tick lineage. However, differences in the salivary gland repertoires and lifestyles of the main families suggest that many blood-feeding mechanisms evolved independently [6]–[8].

Hard ticks are characterized by the presence of a sclerotized scutum, the apical position of their gnathostoma (mouthparts) and numerous denticles on their hypostome [9]. Soft ticks have a leathery integument, nymphs and adults lack a sclerotized scutum and mouthparts are located anterior ventrally [9]. *Nuttalliella namaqua* possess a partly sclerotized pseudo-scutum and an apical positioned capitulum [10]–[11]. However, it also has a leathery integument with few denticles on its hypostome [10]–[12]. It has been described as the “evolutionary missing link” between the hard and soft tick families [10]. Bedford assigned *N. namaqua* to the Ixodidae, related to the genus *Ixodes*, primarily based on the presence of its pseudo-scutum and pre-anal groove [10]. He considered this as evidence for the origins of ixodids in Africa. Schulze and Aragão assigned *N. namaqua* to a separate tick family, the Nuttalliellidae [13]–[14]. Hoogstraal considered the Nuttalliellidae to be a separate truncated branch of the superfamily Ixodoidae that diverged from the Ixodidae close to the last common ancestral node, a notion supported by Oliver [15]–[16]. Recent considerations place the Nuttallielllidae within the Ixodoidea, but leaves the phylogenetic relationships for the three families unresolved, primarily due to the absence of any molecular data for *N. namaqua* [1]–[2]. The latter's phylogenetic position could therefore have significant implications for hypotheses on the evolution of a blood-feeding lifestyle in ticks [7].

The lifestyle strategies of hard and soft ticks and their blood-feeding mechanisms differ significantly [7], [15]–[16]. Hard ticks of all life stages (larvae, nymphs and females) feed for prolonged periods that can last from several days to weeks [9]. They ingest more than hundred times their body weight in blood during feeding and concentrate this blood meal by secretion of excess water (60–70%) back into the host via the salivary glands [17]–[19]. Soft ticks (adults, nymphs and some larvae) feed rapidly to engorgement, within minutes to hours, with the amount of blood taken up limited by the extent that their leathery integuments can expand. This generally results in the uptake of blood two-ten times their initial bodyweight [20]. Excess fluid is secreted by the coxal glands. In the case of *N. namaqua*, no knowledge exists regarding its life stages and feeding habits. It was suggested that the preferential host could be rock hyraxes (*Procavia capensis*), swallows, rodents and meerkat [10]–[11], while *Agama* or other lizards was also considered [15]. Efforts to feed females and nymphs on chickens, pigeons, rabbits, rats or mice were unsuccessful [15]. As yet, no empirical evidence exists to give definitive information on host preference.

Only eighteen *N. namaqua* specimens were found to date in southern Africa and Tanzania. Bedford described the holotype based on one female found under a stone near Kamieskroon, Namaqualand, South Africa [10]. Schulze's tick collection contained a specimen from Windhoek, Namibia [11]. Ten specimens were collected from museum skins of the slender-tailed meerkat (*Suricata suricatta hahni*) from Kobos, Rehoboth district, Namibia and one from Brants' karoo rat (*Parotomys brantsi*), Port Nolloth, Namaqualand, South Africa [21]. Two specimens were collected from the nests of the striped swallow (*Hirundo abyssinica unitatis*) [11]. Most recently, Dixon collected three nymphs and two females on the ground thirteen km south of Springbok, Namaqualand, South Africa in 1980 [22]. Most existing samples are therefore of historic value, more than twenty years old and not useful for DNA extraction as evidenced by previous attempts that failed to obtain adequate quantities for molecular analysis [1]. In this study, new *N. namaqua* specimens were collected to investigate questions regarding its phylogenetic relationships to the other tick families, natural hosts, feeding biology and the evolution of blood-feeding in ticks.

## Results

### Distribution of *N. namaqua*

**Figure 1:**
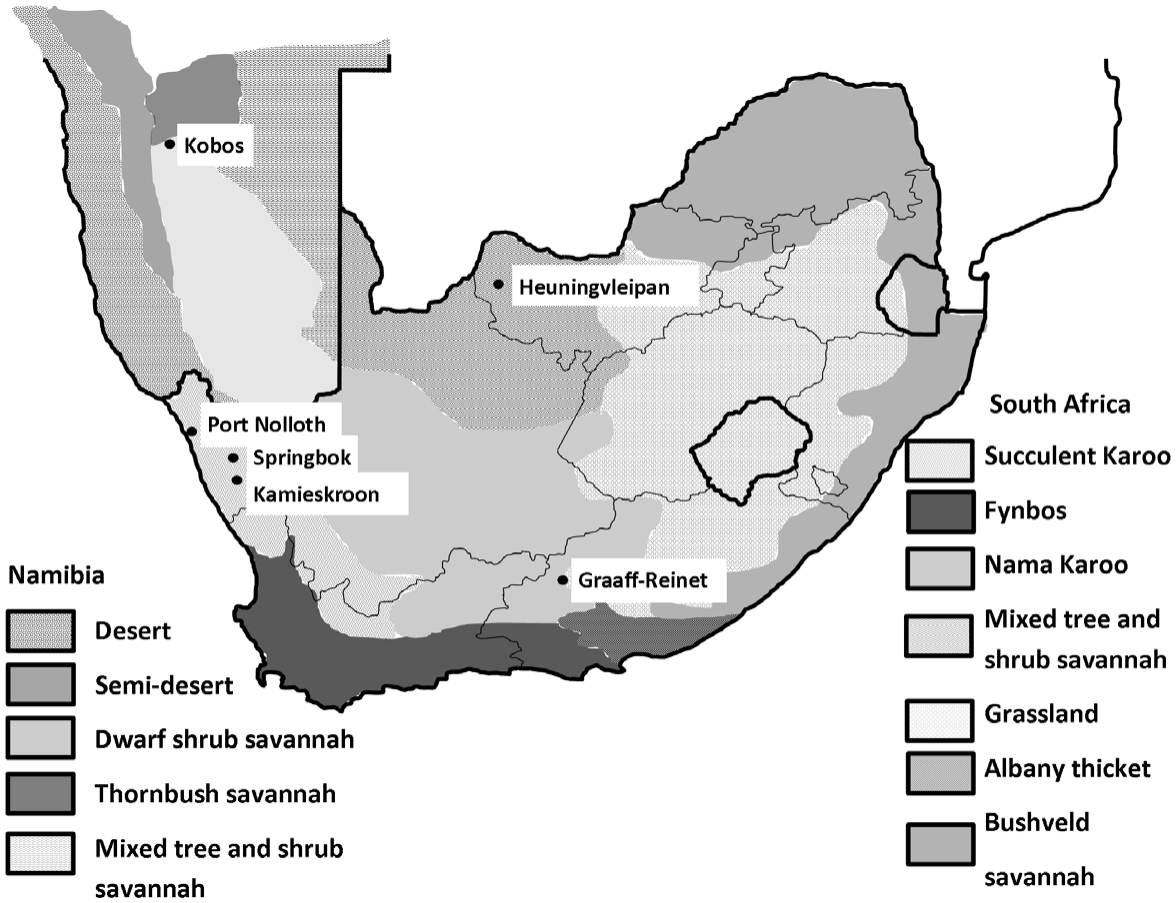
Localities where *N. namaqua* has been collected in southern Africa. Biome data are indicated for Namibia [74], and South Africa [31] and collection sites by black dots and names.

Two new collection localities for *N. namaqua* include Graaff-Reinet in the Eastern Cape (1 nymph) and Heuningvleipan in the North-West province (2 adults) (Fig. 1). Ten live and twenty-one dead specimens were collected near Springbok in Namaqualand, Northern Cape province (Fig. 1). The total specimen count for *N. namaqua* was raised from eighteen females and three nymphs [15], to fifty-one specimens.

**Figure 2:**
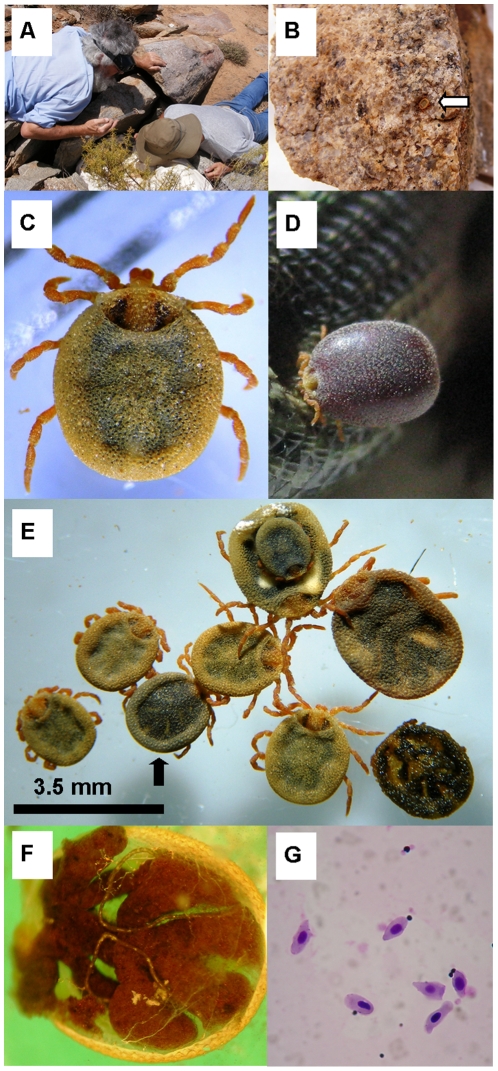
Collection and morphology of *N. namaqua*. A) The crevice from which specimens were collected. B) A specimen concealed on a rock obtained from within the crevice. C) A dorsal view of an unfed female that shows the pseudo-scutum and ventral mouthparts. D) The same tick shown as an engorged female still attached to a lizard. E) Size range and general morphology of the collected live specimens. The black arrow indicates the tick selected for dissection from which lizard DNA was extracted. F) A dissected female with midgut that indicates it's recently fed status. G) A Giemsa stained smear obtained from the gut contents of the dissected female.

Ticks were collected within a rock crevice, clinging to loose rocks wedged inside the fissure (Fig. 2A). Potential vertebrate hosts observed in the vicinity of the collection sites included hyraxes, skinks, elephant shrews, suricates and tortoises. Skinks were abundant in the rock crevice and the Cape skink (*Mabuya capensis*) could be positively identified.

Ticks were identified based on their leathery integument, semi-sclerotized scutum that is wider than it is long, orange legs, presence of ball-joints and apical mouthparts (Fig. 2C and Fig. 2E) [10]. The larvae, not previously described, could be assigned to the *N. namaqua* based on DNA sequencing.

### Dissection of a female tick

A partially-engorged female was dissected (Fig. 2F). The gut shows the typical anterior and posterior stomach lobes with unbranched caeca that is unique to *N. namaqua* [12], [22]. The semi-filled state of the gut indicated that this tick fed in the recent past, but its partial depletion indicated that it has started to process its blood meal (Fig. 2F). Rupture of the gut showed the presence of numerous hematin crystals. A Giemsa stained smear prepared from extruded gut contents showed the presence of intact nucleated red blood cells (Fig. 2G). A second female was dissected, but its gut contents did not show the presence of any intact nucleated red blood cells.

### Identification of previous hosts from the gut content

**Figure 3:**
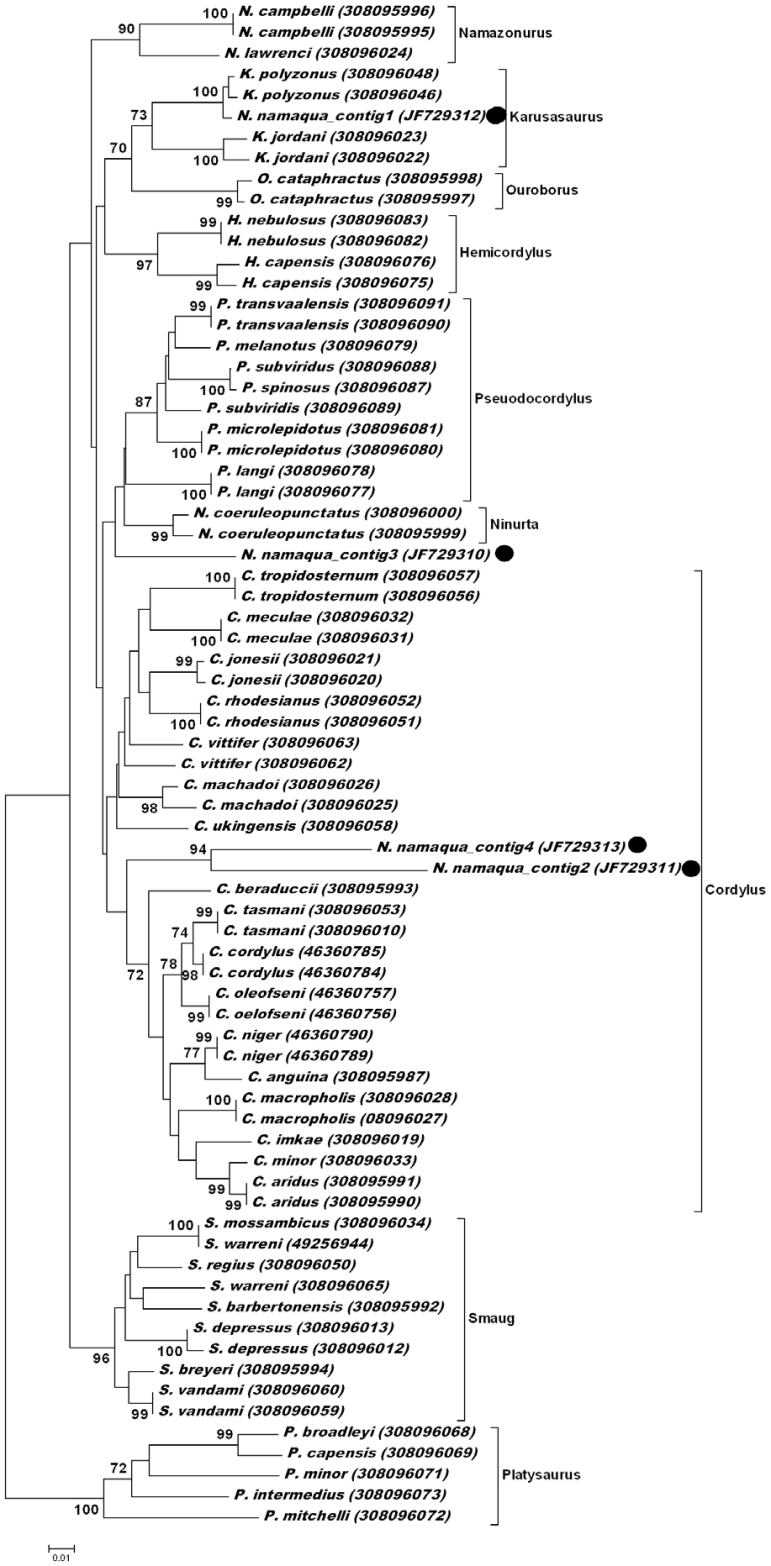
Phylogenetic analysis of the cordylid lizard family and sequences obtained from the gut content of *N. namaqua*. Consensus sequences obtained from *N. namaqua* are indicated with black dots. Clades and genera are labelled according to Stanley et al. [33]. Genbank accession numbers are indicated within brackets.

Nucleated red blood cells in the gut indicated that a previous blood meal was obtained from an avian or reptilian host [23]. Given the collection locality, it was unlikely that birds could be hosts, while numerous lizards were observed at the collection site. However, to ensure detection of both avian and reptilian hosts, primers for the 16S mitochondrial gene that amplifies lizard and avian gene fragments were used [24]. This strategy was followed as it is known that nucleated red blood cells retain mitochondria in lower vertebrates [25]. Sequencing of twenty-six different clones yielded four different 16S rRNA gene fragments (contig 1: 12, contig 2: 5, contig 3: 6, contig 4: 3 sequences, respectively). BLASTN analysis retrieved members of the girdled lizard family (Cordylidae) with E-values of zero. Neighbor-joining analysis indicated that contig 1 group within the *Karusasaurus* clade and possibly represents *Karusasaurus polyzonus*. Contig 2 and 4 grouped with weak support within the *Cordylus* clade with no distinct similarities to any of the lizard sequences currently available in the databases (Fig. 3). Similarly, contig 3 grouped in a clade formed by the genera *Ninurta* and *Pseudocordylus*, but with no distinct similarity to any lizard sequences currently deposited. The gut contents from a second dissected tick did not show any intact red blood cells and no PCR products were obtained from extracted DNA. Neither was any amplification products detected for DNA extracted from larvae.

### Tick feeding

The identification of lizards as potential hosts prompted the feeding of *N. namaqua* on lizards. Both nymphs and adults attached, probed and fed without engorgement. One nymph attached and fed slowly for ∼3 hours before rapid engorgement, which took ∼20 minutes and then remained attached for ∼60 minutes. Four adult females attached and became engorged within 20 minutes. Rapid feeding coincided with rapid expansion of the leathery cuticle as observed for soft ticks (Fig. 2D). A period of slow feeding followed that lasted for 30–120 minutes, during which droplets were expunged from the anal opening and spurts were observed to occur in a rhythmic manner, with an appreciable amount of fluid being secreted (estimated at 30 nl/ 10 seconds). One tick expanded to a fully engorged state in which even the small infoldings of the integument became distended (Fig. 2D). Engorged weights increased ∼5–14 times compared to the unfed weight. The female that engorged to the greatest extent, ingested ∼14 µl final volume (w/v basis), assuming a density of blood of 1.06 g/ml [26]. It also remained attached in the engorged phase for one hour during which time fluid secretion occurred. A secretion rate of 30 nl/ 10 s was calculated based on droplet size excreted, resulting in ∼11 ul of fluid secreted. This would make the final volume of ingested blood ∼25 µl and would indicate that the blood meal was concentrated approximately two fold. Excretion of fluid terminated immediately upon detachment from the host and was not observed for up to an hour after feeding.

### Tick systematics

**Figure 4:**
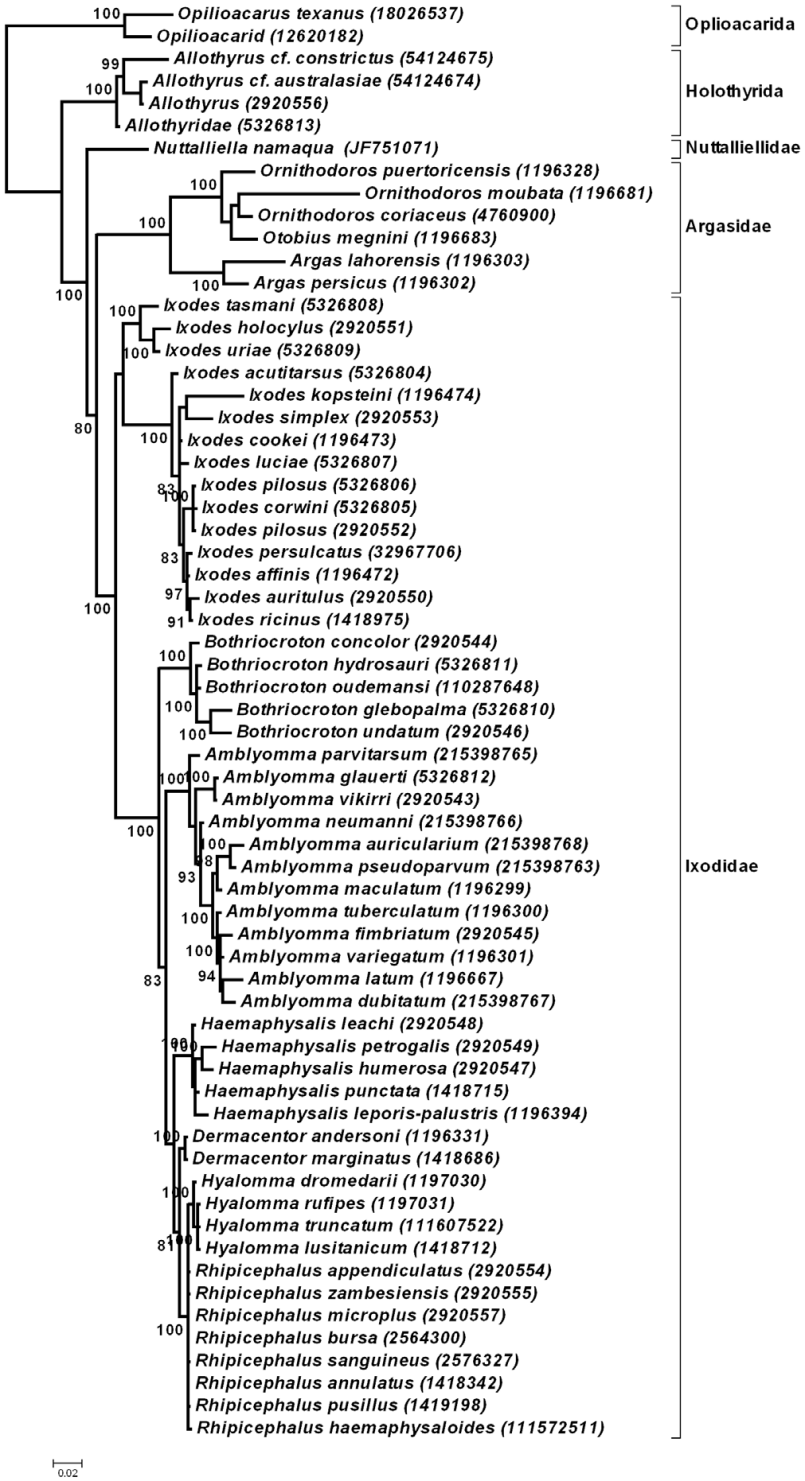
Bayesian analysis of the 18S rRNA gene for the parasitiformes. Nodal support is indicated by posterior probability values. Genbank accession numbers are indicated in brackets.

The small ribosomal nuclear RNA (18S rRNA) gene is the most commonly used molecular marker for the investigation of arthropod and chelicerate relationships at the level of phyla and superphyla [27]–[29]. It was particularly useful in the analysis of phylogenetic relationships within the parasitiform mites and especially ticks at the familial and generic levels [3]–[5]. No nucleotide bias was observed within the 18S rRNA gene fragment (1571 bp) obtained for *N. namaqua* (A 25%, G 27%, C 23%, T 25%) and was comparable to nucleotide frequencies observed for other tick 18S rRNA genes [4]. BLASTN analysis retrieved as best hits members of the hard tick family (E-values = 0) confirming the relationship of *N. namaqua* to the Ixodida. Bayesian analysis of the full 18S rRNA dataset (64 sequences) indicated that the three tick families are monophyletic, but that *N. namaqua* grouped basal to the hard and soft tick families, with the Allothyrida as sister-group (Fig. 4). Most nodes are well supported with posterior probabilities above 95% and the topology of the consensus tree is similar to previous studies for the Ixodida [4]–[5]. This also correlates with general considerations regarding the current knowledge on the phylogenetic relationships for the various tick genera [1], except for the Rhipicephalinae clade which has been condensed due to the fact that this fragment of the 18S rRNA gene is identical for all members.

**Figure 5:**
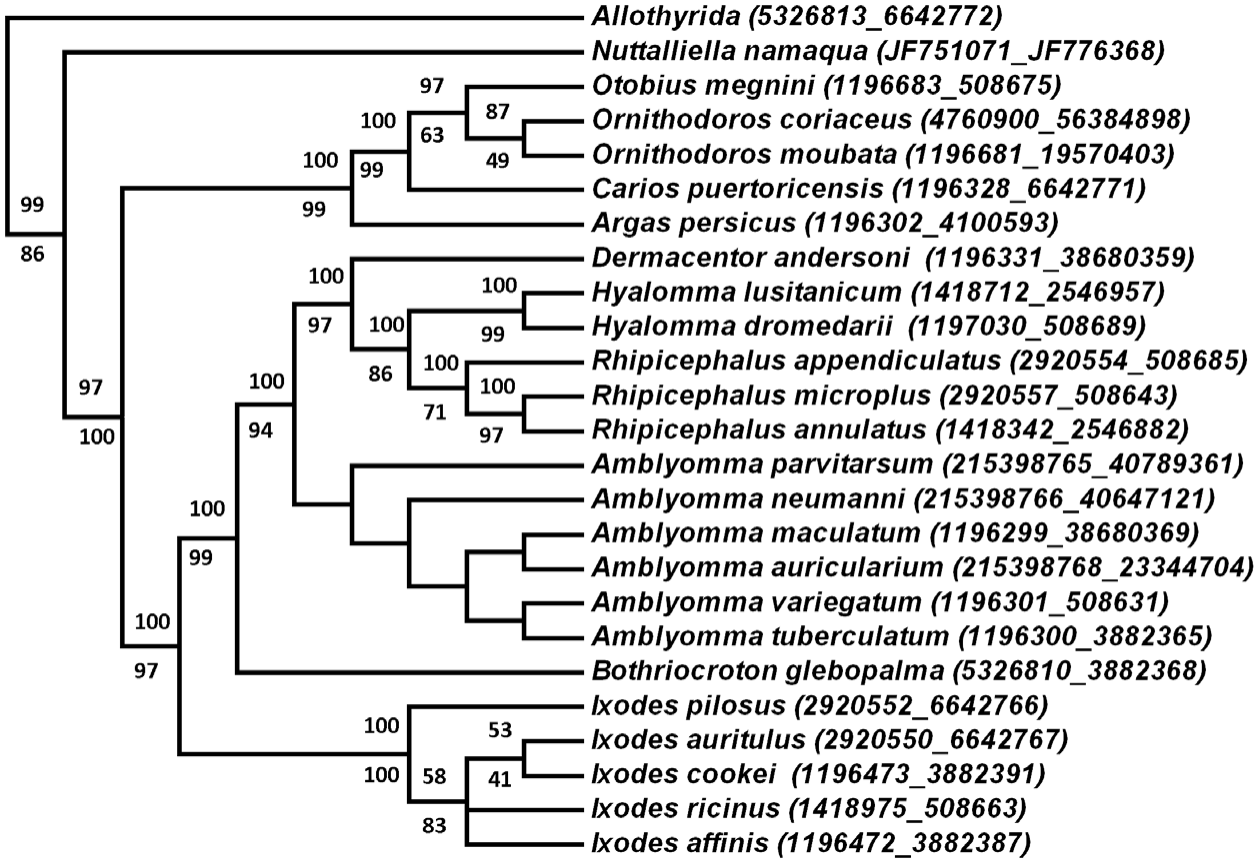
Phylogenetic analysis of the concatenated 18S-16S rRNA dataset. Indicated is the 50% majority consensus tree obtained with Bayesian as well as maximum parsimony analysis. Posterior probability and bootstrap support values are indicated above and below the nodes, respectively. Genbank accession numbers are indicated in brackets as 18S_16S.

The 18S rRNA gene is too conserved to be useful for resolving relationships at lower taxonomic levels and within the Ixodidae many closely related species have 18S rRNA genes with little phylogenetic information [3]. Conversely mitochondrial genes, such as the 16S rRNA gene, are useful to resolve relationships at generic as well as species level and a combination of data could therefore prove to provide phylogenetic signal at both high and low taxonomic levels [3]. We therefore included 16S rRNA data to incorporate fast evolving sites that will allow resolution of closely related species, while retaining the 18S rRNA information necessary to resolve the higher level relationships. This concatenated dataset only contain 25 taxa, but produced similar relationships for the various tick genera and supported the grouping of *N. namaqua* at the root of the tick tree with a posterior probability value of 100 for Bayesian and 97% bootstrap value for maximum parsimony analysis (Fig. 5). Both methods gave consensus trees with similar topologies that recapitulate the current consensus on relationships within the Ixodida at generic level.

## Discussion

The following scenarios for the biology of *N. namaqua*, the origin of ticks and the evolution of blood-feeding behaviour are suggested:

### Geographic range of *N. namaqua*

The geographic range of *N. namaqua* within southern Africa (excluding Tanzania), seems to be distributed across regions primary xeric in nature (Fig. 1). This includes the mixed tree and shrub savannah biome ranging from the Vryburg district in the East to Kobos, Rehoboth in the West, the Nama Karoo biome of the smaller Karoo in the South at Graaff-Reinet and the central succulent Karoo biome of Namaqualand that includes Kamieskroon, Port Nolloth and Springbok [30]–[31]. It is expected that *N. namaqua* will be distributed across the numerous biomes of the greater Karoo area that link these regions (Fig. 1) [30]–[32].

### Natural hosts for *N. namaqua*

The identification of 16S rRNA genes of four different lizard genotypes from the blood meal suggests that this female fed at least four times. The blood meal can therefore be stored for prolonged periods of time with adults feeding several times intermittently. It also suggests that the preferred natural hosts are lizards. It is not known whether larvae and nymphs would preferentially feed on lizards or whether elephant shrews or rodents could be possible hosts. Previous records of *N. namaqua* obtained from museum skins of rodents and suricates as well as from bird nests [11], could indicate that it is a generalist and that its host preferences depends on its ecological habitat. This can as yet not be excluded, although the successes described in the feeding of *N. namaqua* on lizards suggest that they might be preferential hosts.

All host DNA identified belong to the Cordylus family (80 named taxa) of scinciform lizards that is endemic to sub-Saharan Africa [33]. *Karusasaurus* (2 species) are limited to semi-arid areas in South Africa and Namibia, while *Cordylus* (20 species) is widely distributed from South Africa as far north as Ethiopia [33]. Most members are highly adapted to rock-dwelling lifestyles and would therefore fill potential ecological niches for *N. namaqua*. The wide distribution of this lizard family, linked with the finding of *N. namaqua* in Tanzania could suggest that the Nuttalliellidae could be much wider distributed than the current data suggests.

### Feeding, blood meal processing and concentration

Previous attempts to feed *N. namaqua* on a variety of vertebrate hosts were unsuccessful and included chickens, pigeons, hamsters, rabbits, mice or rats [12], [15]. Successful feeding of all selected nymphs and adults on lizards were therefore significant.

The long periods of attachment without feeding have been observed in many argasids [34]. More noteworthy is the rapid engorgement (10–30 minutes) followed by slow engorgement for up to an hour. This slow phase probably occur to concentrate the blood meal, which were estimated to be approximately two fold. This correlates well with values estimated for argasid ticks [20]. A packed cell volume of 37% was determined for the lizards used for feeding, which correlated with packed red blood cell volumes determined for other lizards (30–40%) [35]. A 2–3 fold blood meal concentration would be close to the limits of blood concentration that can be expected in the absence of cuticular growth as observed for ixodids and the absence of red blood cell lysis.

No secretion of coxal fluid was observed either during feeding or after detachment and was confirmed over a period of several days. This contrasts with argasids, which secrete coxal fluid during feeding or after detachment [34], [36]. This was not due to limited engorgement, as the tick was fully distended after repletion. In addition, no evidence could be found for coxal organs in two dissected ticks and corresponds to previous observations [22]. Active secretion of fluid from the anal pore was observed during feeding and occurred most probably via the malphigian tubules, suggesting that the coxal organs are absent in *N. namaqua*. Secretion of nitrogen waste occurred once the blood meal was assimilated by the ticks and could be observed as white guanine deposit.

Excretion of fluid during and after a blood meal, by malphigian tubules via the rectal ampulla, has been observed in the soft tick, *Ornithodoros moubata*, which do not defecate due to a blind hindgut [37]. This mode of fluid secretion is therefore considered to be ancestral, with independent evolution of fluid secretion via the coxal organs and salivary glands in the respective tick families.

In ixodids, red blood cells are rapidly lysed after ingestion and released haemoglobin taken up by digestive cells and stored in endosomes until proteolytic digestion in the lysosomes [17]. In contrast, red blood cell lysis occurs in two phases within argasid ticks [17], [20]. Initially, some red blood cells are lysed after detachment and the released haemoglobin stored within the gut in crystalline form before being taken up by endocytosis for proteolytic digestion. The remainder of the red blood cells are stored in the caeca in an unlysed form [20]. Feeding, blood meal storage and possibly digestion in *N. namaqua* is therefore similar to argasids and we assume that the argasid mode of feeding is ancestral, as proposed by numerous authors that assumed the argasid-lineage to be more primitive [15]–[16], [38]. The absence of intact red blood cells in the second dissected tick could indicate that blood meal digestion has progressed to a stage where all blood cells have been lysed. It could also indicate that this tick has mated after it obtained its previous blood meal [20]. Alternatively, this tick could have fed on a mammalian host and would therefore not possess any nucleated red blood cells.

### Ancestral morphological features of *N. namaqua*

The basal position of *N. namaqua* in the tick tree suggests several interpretations for morphological features shared with the main tick families or considered to be unique to *N. namaqua*. The presence of a pseudo-scutum or true scutum in ticks would be a derived ancestral parasitiform character and fits with the observation that scutums are also prevalent in holothyrid and larval argasids [39]. In ixodids this character became prominent due to its excellent protective features during their prolonged periods of host association. Bedford considered *N. namaqua* to be closest related to the *Ixodes* based on the existence of a pre-anal groove [10]. However, pre-anal grooves are also present in the Ornithodorinae and have been considered to be an ancestral character of the Ixodida [39]. The statement that the gnathostoma of *N. namaqua* have an apical position [11], should be tempered by the description of Bedford [10], that indicated a very short base dorsally and elongated ventrally. When the photographs of the current study is scrutinized it is clear that while the gnathostoma can be seen from the dorsal side (similar to ixodids), it is in fact located apical-ventrally (similar to argasids). Its intermediate position is similar to that observed for the holothyrida, but lacks the distinct camerostome found for holothyrida as well as the Argasidae [40]–[41].

### The origins and hosts of the ancestral tick lineage

Considerations on the origins of ticks span almost 300 million years and ranges across many evolutionary epochs that include: the late Silurian (443–417 million years ago - MYA) [42], Devonian (417–362 MYA) [16], [43], late Permian (290–248 MYA) [44], Triassic (248–206 MYA) [15], [45]–[46] and Cretaceous (146–65 MYA) [3], [47]–[49]. Recent views consider the origins of ticks to have occurred in Australia or its counterpart of the Gondwanan landmass, either in the Devonian (390 MYA) [43], or the Late Cretaceous (120 MYA) [3], [49]. The former was based on a consideration of the limited geographic distribution of the extant Holothyrida to Australasia [1]. The three families found within the Holothyrida are, however, more widely distributed than Australasia and has been found in the New World, with suspected current distributions that might extend to Madagascar and the mountains of East Africa [50]–[51]. Klompen considered that the origins of the Australian ixodid lineages, many basal within the Ixodidae, could only have occurred after the breakup of Gondwanaland and by extension the rest of the ixodid family [3].

Given the basal position of *N. namaqua* in relation to the major tick families, this species is the closest living relative to the last common ancestral lineage. Its limited distribution to southern Africa makes a good case for the origins of ticks in this region of Gondwanaland. This extends the suggestion of Bedford for the origins of the Ixodidae in Africa to the Ixodoidea [10]. Recent molecular clock estimates, as well as paleontological considerations would place the origin of parasitiform mites and ticks close to the Late Carboniferous/ Early Permian (300±27 MYA) [52]–[53]. This is an interesting period in the evolution of vertebrate life in southern Africa, specifically the Karoo basin [54]–[55]. In the Karoo the ideal climatic conditions for the radiation of ectothermic tetrapods were established during the middle Permian (270–260 MYA), when climate shifted from ice-house to hot-house conditions [54]. This period saw the evolution of the numerous therapsid lineages (synapsid mammalian-like reptiles) in the Karoo that eventually gave rise to mammals [54]. The largest global mass extinction event occurred at the end of the Permian (Permo-Triassic Boundary – 251 MYA) with subsequent recovery and diversification of numerous vertebrate taxa in the Karoo basin [56]–[57]. In this regard, fossil evidence indicates that diapsid reptiles only appeared in the Karoo basin in the Triassic, probably due to migration from other geographic regions [54], [58].

We propose that the ancestral tick lineage originated in the middle Permian (260–270 MYA) in the Karoo-basin and parasitized therapsids. The diversification observed for vertebrates and particularly diapsid reptiles in the Karoo basin after the Permian mass extinction was paralleled by the speciation events that gave rise to the main tick families in the Triassic. It also suggests that the Ixodida narrowly escaped extinction.

It is possible that the Permian mass extinction event saw a decline in species richness of the Nuttalliellidae due to host decimation. The paucity of extant species richness could therefore be due to the fact that *N. namaqua* is a monotypic “dead clade walking” and therefore a living fossil [59]. The relative success of the main tick families with regard to species richness would therefore be due to their ability to have adapted and diversified with their respective hosts and varied ecological habitats. The origins of ticks in the Karoo could also explain why *N. namaqua* remained a living fossil, since the basic ecology of the Karoo has remained constant since Late Permian times, when climatic conditions in the Karoo basin turned from a relative wet cool climate to semi-arid conditions [60]. This would have been exacerbated by its proclivity to inhabit rock crevices that would maintain sheltered micro-habitats that are frequented by small crevice crawling lizards.

The only major therapsid lineage that survived the End-Permian extinction was *Lystrosaurus* (95% of all early Triassic terrestrial fossils) and its ability to survive far into the Triassic at low diversity (two species) was linked to it taking refuge in burrows [56], [61]. It is therefore possible that *N. namaqua* parasitized this lineage and when the therapsid lineages were replaced mostly by diapsids [54], [56], [58], host switching occurred and lizards became preferential hosts. Similarly, lizards were probably some of the major host species parasitized by hard and soft ticks, until mammals and birds supplanted them as hosts. Association with synapsid reptiles and their dispersal across Gondwanaland [54] could have triggered longer host association that eventually manifested in the typical life cycle of ixodids.

### Implications for the evolution of salivary gland protein complexity

The implication of the current study is that blood-feeding behaviour evolved within the ancestral tick lineage, before divergence into the main tick families. This fits parsimonious arguments for the origins of blood-feeding behaviour in ticks, given that all ticks are obligate blood-feeding ecto-parasites. This is in contrast to the proposal that the hard and soft tick families evolved blood-feeding behaviour independently [6]–[8]. The latter proposal was based on the extensive differences observed in salivary gland sialomes of the hard and soft ticks. In this regard, few orthologs with conserved function are conserved between the tick families [62]. It was shown that although the ancestral tick lineage would have possessed the major salivary gland protein families, most of the gene duplications found in these families are lineage specific expansions, indicating that functions associated with these occurred after divergence of the main tick families [62]. How can these differences be reconciled with the conclusion that all ticks share a common blood-feeding ancestral lineage?

The proposed origins for ticks in the Late Permian (260–270 MYA) occurred just before the End Permian extinction event (251 MYA), while the main tick families, speciated in the Early Triassic (240–230 MYA). The origins, adaptation to blood-feeding and speciation therefore happened over a short period of time that was marked by its own turbulent history of therapsid origins, extinction and expansion of new vertebrate host species. Concurrent adaptation to blood-feeding linked with host switching during this period could have played a major role in the evolution of blood-feeding behaviour of the main families. This probably had a more significant effect in ixodids, due to their longer association with their hosts. In contrast, soft ticks feed fast and it has been shown that their hemostatic and immune-modulatory systems have been conserved in the major argasid genera even though these feed on birds and mammals, respectively [63]–[64]. Thus, even though blood-feeding evolved in the ancestral tick lineage, the adaptation to the mammalian and avian blood-feeding interfaces occurred independently in the soft and hard tick families. It would be of interest to determine whether the anti-hemostatic and anti-inflammatory mechanisms conserved in soft ticks, has been present in the ancestral tick lineage and whether these would be found in *N. namaqua*. From a comparative analysis of salivary gland transcriptomes of hard and soft ticks it was shown that all of the major protein families are conserved, but that the majority of gene duplications are lineage specific expansions that occurred after the divergence of the hard and soft tick families [62]. This suggested that the ancestral tick lineage had a simple (few members for each family), but diverse (many different protein families) salivary gland protein domain repertoire. In regard to a reconstruction of ancestral proteins evolved for tick-host interaction, a common blood-feeding origin allows for the assignment of various proteins found in hard and soft tick salivary glands to ancestral evolved functions. These would include tick apyrases and biogenic amine-binding proteins [65]–[66]. The testing of these hypotheses would be possible once the sialome for *N. namaqua* has been determined.

### Conclusions

In conclusion, phylogenetic analysis indicates that *N. namaqua* groups basal to both tick families and is the closest extant lineage to the last common ancestral tick lineage. Its argasid-like feeding behaviour and biology provides compelling evidence for the evolution of a blood-feeding lifestyle within the last common ancestral tick lineage. The semi-arid nature of the Northern Cape as found in Namaqualand and the Karoo has been maintained since Permian times. The partiality of *N. namaqua* for xeric environments and small reptiles could therefore be an indication of a lifestyle maintained for more than 250 million years. This would truly make this tick species a living fossil.

## Materials and Methods

### Ethics statement

All experiments related to the lizard feedings were performed in strict accordance with the Ethics guidelines from the Onderstepoort Veterinary Institute. Experiments were approved by the Onderstepoort Veterinary Institute Animal Ethics Committee (approval number: AEC12.11) and falls under the routine tick feeding and colony maintenance project.

### Tick collection

Ticks were collected at Krymekaar (S29°46.033′; E 017°50.491′) and Voëlklip (S29°44.518′; E 017°51.769′), ∼13 km south of Springbok, Namaqualand, South Africa in the proximity where Dixon collected *N. namaqua* specimens in 1980 [22]. All necessary collection and transport permits were obtained from the Veterinary Authorities (Permit number: SP2011/02/02/01). In addition permission to collect ticks from Krymekaar and Voëlklip was granted by the owner, Mr. A. van Heerden. A single female collected near Krymekaar were brushed from the roof of a rock crevice habited by hyraxes (*Procavia capensis*). Two live nymphs and seven adults as well as two dead nymphs, six dead adults and twelve dead larvae were collected near Voëlklip from a rock crevice in the ground habited by lizards and elephant shrews (undetermined species). In addition, two females were collected near Heuningvleipan, North-West province (1991) on a rock wall and one nymph in a collapsed eagle nest near Graaff-Reinet (1995), by one of the authors (DdK). Ticks were deposited in the Onderstepoort Tick Museum under the collection numbers OP3403–OP3409.

### Tick dissection, preparation of blood smears and DNA extraction

Two female ticks were embedded in wax and the dorsal cuticles removed using a scalpel under phosphate buffered saline to reveal the undisturbed gut. Guts were removed and contents extruded to prepare Giemsa stained smears and the remainder used for DNA extraction. The carcasses were extracted separately for DNA. In addition, four dead larvae were pooled and DNA extracted. All DNA extractions were performed using the Roche MagnaPure (Roche Diagnostics) and the MagNa Pure Large Volume DNA Isolation Kit (Roche Diagnostics).

### Amplification and sequencing of the 16S mitochondrial and 18S nuclear tick DNA

The tick 18S rRNA fragment was amplified with high fidelity KAPA long range polymerase (KapaBiosystems Inc, Woburn MA, USA) using the 18S NS1 and 18S NS8 primer set [67]. PCR products were cleaned up using the silica clean-up kit (Fermentas) and sequenced using the BigDye® Terminator v3.1 Cycle Sequencing Kit (Applied Biosystems) with the 18 NS1 primer as well as internal primers to obtain a 1571 bp consensus sequence for the 18S rRNA gene. At least four separate PCR products were cloned and sequenced for every tick sample. The 16S rRNA gene was amplified using *Pfu* polymerase (Fermentas) with the 16S+1 and 16S−1 primers [48]. At least four separately amplified PCR products were cleaned up and sequenced from both sides using the same primers to obtain a 454 bp consensus sequence.

### Bioinformatics for the tick 18S nuclear gene

Sequences for the tick 18S rRNA genes were extracted from Genbank using BLASTN analysis [68]. All tick sequences, and sequences for Opilioacarida (outgroup) and Allothyrida, were extracted and edited to yield a non-redundant dataset of 64 sequences. Sequences were aligned using a consideration of the secondary structure of RNA (Q-INS-i) as implemented in MAFFT [69]. Alignments were manually inspected, adjusted and edges trimmed to give 1610 aligned characters of which 264 were phylogenetic informative sites.

Bayesian analysis was performed using MrBayes 3.1.2 [70], using a general time reversible (GTR) of nucleotide substitution with a proportion of invariant sites and a gamma distribution of among site heterogeneity using the nst = 6 rates = ingamma command. Four categories were used to approximate the gamma distribution and two runs were performed simultaneously, each with four Markov chains (one cold, three heated) which ran for 3,000,000 generations. The first 300 000 generations were discarded from the analysis (burnin) and every 100^th^ tree was sampled to calculate a 50% majority-rule consensus tree. Nodal values represent the posterior probability that the recovered clades exist, given the sequence dataset and are considered significant above 95% [28].

For the 16S rRNA gene, sequences for which 18S rRNA genes from the same species are available in the database were extracted to yield a non-redundant dataset of 25 sequences that included one sequence for the Allothyrida (outgroup). These sequences were concatenated with the 18S rRNA gene, aligned as above and the most variable regions (gapped positions) within the 16S rRNA gene removed to produce an alignment of 1822 bp with 290 phylogenetic informative sites. Bayesian analysis was performed as described above, while maximum parsimony analysis for this dataset was performed using the Mega4 program [71]. For maximum parsimony, all sites were used and a tree search were performed using close-neighbor-interchange (search level = 1) and random addition of trees of 500 replications. Nodal support was estimated using bootstrap analysis (10 000 replicates).

### Amplification and sequencing of the 16S lizard mitochondrial DNA

Primers for the 16S rRNA gene of sub-Saharan scincine lizards were selected based on the identification of the common Cape skink (*Mabuya capensis*) near the Voëlklip collection site. The 16S rRNA gene (∼600 bp) was amplified using the 16S F.1 and 16S R.0 primer set [24], with *Pfu* polymerase (Fermentas). BLAST analysis of this primer set indicated that it will also amplify the 16S rRNA gene from other reptiles and birds. PCR products were cloned into the pGEM T-Easy vector system and colonies screened using the M13 vector primer set. Twenty six positive colonies were cleaned up and sequenced with the M13 reverse primer using the BigDye® Terminator v3.1 Cycle Sequencing Kit (Applied Biosystems). Four different 16S rRNA consensus sequences were obtained from the 26 clones sequenced and sequences were submitted to Genbank (Liz1: JF729312; Liz2: JF729311; Liz3: JF729310, Liz4: JF729313).

### Bioinformatics for the lizard 16S mitochondrial gene

The 16S rRNA lizard sequences were analysed against the non-redundant database using BLASTN analysis [68]. For each, the first hundred best hits were retrieved, combined and filtered to give a non-redundant master sequence data set of 139 sequences. In the case where multiple sequences from a specific species were obtained, the number of specimen sequences was limited to two. As outgroup, members of the genus *Platysaurus* were included [33], to give a final master set of 73 sequences that were aligned using ClustalX [72].

Alignments were manually inspected, adjusted and edges trimmed to give 387 bp of which 128 were phylogenetic informative sites. Neighbor-Joining analysis was performed using Mega4 software with the Tamura-3 paramater model [71]. Gaps were treated as pairwise deletion and both transitions and transversions were included in the analysis. Rates among sites were treated as uniform and patterns among lineages as heterogeneous. Branch support was estimated using bootstrap analysis (10 000 replicates).

### Tick feeding on lizards

Skinks (*Mabuya* genus) captured at Onderstepoort Veterinary Institute was used for tick feeding. Lizards were restrained by hand and ticks were allowed to roam freely until attachment. Lizards were then kept immobile by hand and feeding observed under a stereomicroscope until completion, after which they were released. Packed cell volumes were determined by collecting blood in capillary tubes [73].
